# Genomic trajectory in leukemogenesis of myeloproliferative neoplasms: a case report

**DOI:** 10.1186/s12920-021-00986-z

**Published:** 2021-05-22

**Authors:** Yujie Chen, Rafee Talukder, Brian Y. Merritt, Katherine Y. King, Marek Kimmel, Gustavo Rivero, Romina Sosa

**Affiliations:** 1 Department of Statistics and Bioengineering, Rice University, 6100 Main Street, Houston, TX USA; 2 Department of Medicine, Baylor College of Medicine, 1 Baylor Plaza, Houston, TX USA; 3 The Dan L. Duncan Comprehensive Cancer Center at Baylor College of Medicine, 1 Baylor Plaza, Houston, TX USA; 4 Fox Chase Cancer Center, 333 Cottman Ave, Philadelphia, PA 19111 USA; 5 Department of Pathology and Immunology, Baylor College of Medicine, 1 Baylor Plaza, Houston, TX USA; 6 Department of Molecular and Human Genetics, Baylor Genetics and Baylor College of Medicine, 1 Baylor Plaza, Houston, TX USA; 7 Department of Pediatrics, Section of Infectious Disease, Baylor College of Medicine, 1102 Bates St. Suite 1150, Houston, TX USA; 8 Section of Hematology and Oncology, Baylor College of Medicine, 1 Baylor Plaza, Houston, TX USA

**Keywords:** Essential thrombocythemia, Leukemia, Clonal evolution, Myeloproliferative neoplasms, Case report

## Abstract

**Background:**

We report a patient with Essential Thrombocythemia (ET), subsequently diagnosed with concurrent myeloid and lymphoid leukemia. Generally, the molecular mechanisms underlying leukemic transformation of Philadelphia-negative myeloproliferative neoplasms (Ph-MPN) are poorly understood. Risk of transformation to acute myelogenous leukemia (AML) is low; transformation to both AML and acute lymphoblastic leukemia (ALL) is extremely low. Genetic defects, including allele burden, order of mutation acquisition, clonal heterogeneity and epigenetic mechanisms are important contributors to disease acceleration.

**Case presentation:**

A 78-year-old Caucasian female originally treated for stable ET, underwent disease acceleration and transition to myeloid sarcoma and B-cell ALL. Genomic reconstruction based on targeted sequencing revealed the presence of a large del(5q) in all three malignancies and somatic driver mutations: *TET2, TP53*, *SF3B1*, and *ASXL1* at high allele frequency. We propose that the combination of genetic and molecular abnormalities led to hematopoietic stem cell (HSC) injury and disease progression through sub-clone branching. We hypothesize that ancestral reconstruction of genomic data is a useful tool to uncover subclonal events leading to transformation.

**Conclusions:**

The use of ancestral reconstruction of genomic data sheds light on the unique clinical scenario described in this case report. By determining the mutational profile of tumors at several timepoints and deducing the most parsimonious relationship between them, we propose a reconstruction of their origin. We propose that blast progression originated from subclonal events with malignant potential, which coexisted with but did not originate from *JAK2 p.V617F*-positive ET. We conclude that the application of genomic reconstruction enhances our understanding of leukemogenesis by identifying the timing of molecular events, potentially leading to better chemotherapy choices as well as the development of new targeted therapies.

## Background

The molecular mechanisms underlying leukemic transformation of Philadelphia-negative myeloproliferative neoplasms (Ph-MPN) are poorly understood. Risk of transformation to acute myelogenous leukemia (AML) is approximately 10–20% for patients with primary myelofibrosis (PMF) and considerably lower for those with Polycythemia Vera (PV) and Essential Thrombocythemia (ET), 5–10% and 2–5% respectively [1, 2]. Transformation of MPN to acute lymphoblastic leukemia (ALL) is rare, with only seventeen cases reported in the literature [3]. Retrospective studies suggest that genetic defects, including allele burden, order of mutation acquisition, clonal heterogeneity and epigenetic mechanisms play an important role in the observed conversion rate, as well as the varied clinical-pathologic entities that evolve [4, 5].

In this report, we describe a series of clonal events in an elderly patient originally diagnosed with *JAK2 V617F*-positive ET who presented in accelerated phase, with subsequent progression to concurrent myeloid sarcoma (MS) and B-cell ALL. We hypothesize that reconstruction of genomic mutations to build an ancestral tree may be a useful tool to characterize leukemogenesis in patients with high-risk disease.

## Case presentation

A 78-year-old Caucasian female was diagnosed with *JAK2* V617F-positive ET in 2010 based on laboratory data and molecular profiling of peripheral blood. She was initiated on hydroxyurea (HU) and low-dose aspirin. The patient remained on anticoagulation with coumadin due to a prior history of atrial fibrillation. She demonstrated stable and adequate platelet response to 10 mg/kg of HU.

**Fig. 1:**
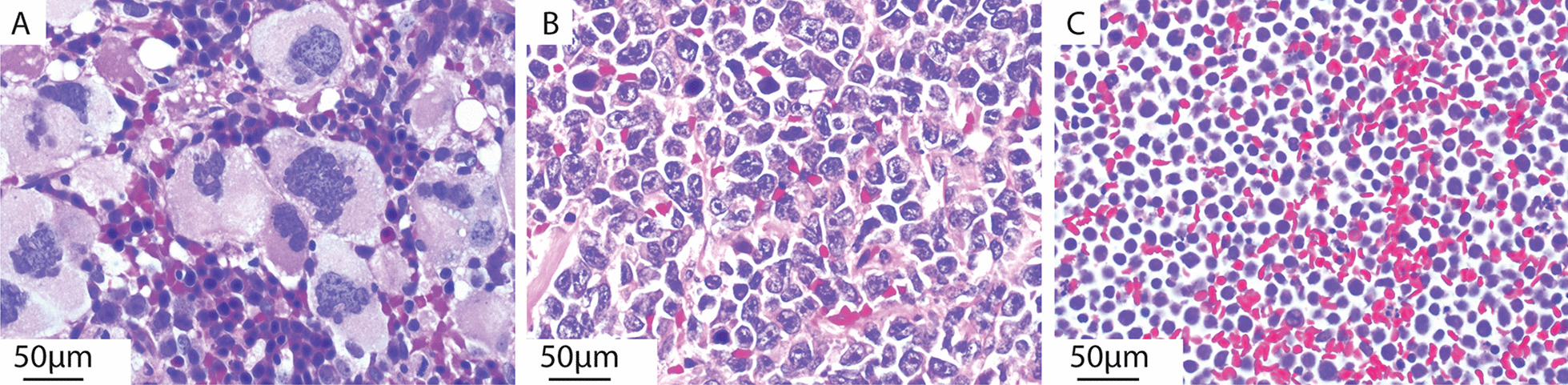
Summary of stages of leukemogenesis in the same patient. Tissue and cell-block formalin-fixed paraffin embedded (FFPE) sections stained with hematoxylin and eosin (H&E) were visualized by conventional bright-field Kohler illumination light microscopy (Olympus BX40, Japan) with a 20 × Plan achromat objective (Olympus, Japan), 20 × camera mount lens and a DP71 camera (Olympus, Japan). Photomicrographs were obtained after white balancing using the CellSense software (Olympus, Japan). Minor contrast adjustments were performed to the entire image to accurately represent the cells observed in manual microscopy. **a** Bone marrow biopsy prior to lenalidomide in 2016 at time of accelerated ET, with abnormally increased numbers of megakaryocytes that are atypical with hyperlobation, nucleomegaly and nuclear hyperchromasia and nucleoli with surrounding myeloid and erythroid progenitors. H&E nominal field magnification 400X. **b** Biopsy of ileocecal mass at time of leukemic transformation in 2018 diagnostic for myeloid sarcoma with sheets of MPO-positive myeloblasts with scant eosinophilic cytoplasm, irregular nuclear contours and prominent nucleoli. H&E nominal field magnification 400x. **c** Pleural fluid aspirate cytology cell block at time of leukemic transformation in 2018 demonstrates numerous blasts with scant cytoplasm and indistinct nuclear chromatin and surrounding red blood cells. H&E nominal field magnification 400X. Scale bars 50 µm

In 2016, despite compliance with therapy, she presented with platelet count of 1150 k/µl, hemoglobin of 8.2 g/dL, and WBC of 6.0 k/µl with absolute neutrophil count of 2820/µl without detectable peripheral blasts. She underwent a bone marrow biopsy, which revealed 80–90% cellularity with increased myeloblasts (12%), megakaryocyte hyperplasia, dysmegakaryopoiesis and grade 2/4 reticulin fibrosis (Fig. 1a). Cytogenetics revealed del(5)(q22-q33),del(17)(p11.2)[17]/46,XX[3], with wild-type *CALR* and *MPL*. Fluorescent in situ hybridization (FISH) reconfirmed 5q deletion. Next generation sequencing (NGS) revealed the following mutations: *JAK2* p.V617F, *SF3B1* p.R625C and *TET2* p.C1271fs (Table 1). Due to patient preference for an oral agent, lenalidomide was initiated at 10 mg orally daily for 21 days in a 28-day cycle. This intervention resulted in partial remission based on International Working Group-Myeloproliferative Neoplasms Research and Treatment Response Criteria, with clinical improvement of anemia and thrombocytosis (hemoglobin of 12.1 g/dL and platelet count of 344 K/µL, respectively). After seven cycles of lenalidomide, a repeat bone marrow biopsy revealed persistent blasts at 8%, persistent dysmegakaryopoiesis, and stable *JAK2* p.V617F mutation. Despite cytogenetic remission, (46 XX in 20 metaphases analyzed), her MDS FISH panel revealed a new 20q12 (PTPRT/MYBL2) loss.

**Table 1:** Summary of Karyotype, FISH and molecular mutations

Disease stage		Peripheral blood 2010	Bone marrow 2016	Bone marrow 2017	Bone marrow 2018	Colonic mass 2018	Pleural effusion 2018
		Essential Thrombocytosis	Accelerated Phase Essential Thrombocytosis	Accelerated Phase Essential Thrombocytosis		Myeloid Sarcoma	B-cell ALL
*Chr*	*Mutations*						
1	NRAS (NM_002524) c.34G > A (p.G12S)		0.0			73.3	0.0
2	SF3B1 (NM_012433) c.1873C > T (p.R625C)		34.7			43.5	47.6
4	TET2 (NM_001127208) c.3812dupG (p.C1271fs)		33.8			47.9	48.4
9	JAK2 (NM_004972) c.1849G > T (p.V617F)	Pos	35.2	Pos		0.0	0.0
17	TP53 (NM_000546) c.215C > G (p.P72R)		45.5			9.3	3.4
17	TP53 (NM_000546) c.734G > C (p.G245A)		0.0			83.7	93.8
1	MPL ex. 10		Neg	Neg		Neg	Neg
19	CALR ex. 9		Neg	Neg			
	*Karyotypes*						
	5del (q22-q33)		43.5		0.5	43.0	98.5
	17p del		85.0			49.0	
	20q del			9.0	28.5		96.0
	MLL (KMT2A) amplification				0.0	74.0	92.0
	MYC amplification						21.5

Variant allele frequencies are defined as fractions of variant versus total sequencing read count expressed as percentages. Frequencies of chromosomal abnormalities are estimated similarly

*FISH* fluorescent in situ hybridization, *ALL* acute lymphoblastic leukemia

**Fig. 2:**
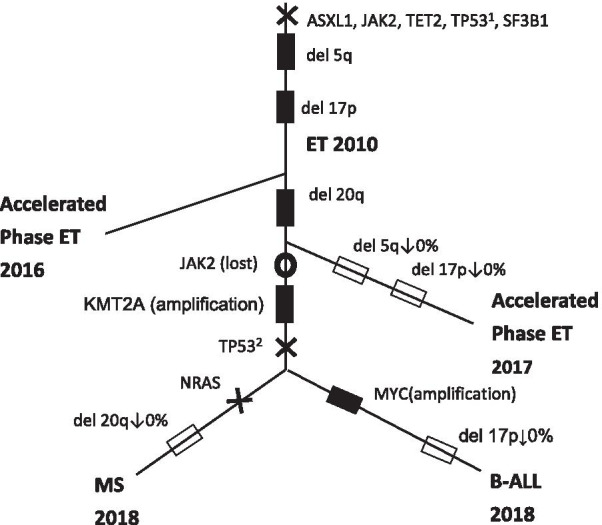
Inferred phylogenetic tree of mutational process contributing to the sequential appearance of MDS/ET, MS and B-cell ALL. Branch splits are consistent with NGS and FISH data reviewed in Table 1. *Notation:* Chromosomal aberrations are denoted by a square. Solid squares (filled square) denote presence of chromosome abnormality; open squares (open square) denote their disappearance. X marks denote presence of point mutations, while open circles (circle) denote loss of mutation. The order of mutations between branch splits cannot be inferred from the data and therefore they are listed lexicographically. The founder cell is proposed to be an HSC containing del (5q) as well as somatic driver mutations (*ASXL1, JAK2, TET2, TP53, SF3B1*). Prior to blast transformation, the pluripotent HSC capable of myeloid and lymphoid differentiation, acquired new *KMT2A* (MLL) amplification. Absence of MLL amplification in bone marrow suggests this clone seeded into the periphery thereby accounting for its presence in the extramedullary blast transformation but not in the bone marrow. Deletion 17p was present in 2016 accelerated phase ET and appears to have been conserved in the subclone that evolved into myeloid sarcoma. Deletion 20q was first seen in bone marrow after lenalidomide therapy and is present in subclone that evolved into B-cell ALL. Treatment with lenalidomide contributed to the suppression of del(5q) clones in bone marrow but had little effect in extramedullary leukemias

In January 2018, she presented with shortness of breath, abdominal pain, nausea and vomiting. Computed tomography (CT) scan of chest, abdomen and pelvis revealed a large 7.9 cm × 4.9 cm ileocecal mass inducing partial colonic obstruction and moderate left pleural effusion. Pathology of the excised cecal mass showed a cluster of medium-sized MPO-positive myeloblasts with eccentric nuclei and scant cytoplasm, positive for CD33 and CD117 based on flow cytometry and consistent with MS (Fig. 1b). Macroscopic examination of her pleural fluid revealed medium-sized cells with large nuclei and scant basophilic cytoplasm positive for CD19, TdT and CD20 consistent with B-cell ALL (Fig. 1c). NGS of both the colonic mass and pleural fluid revealed *SF3B1*, *TP53* exon 6 and exon 3 and *TET2* (Table 1). *JAK2-V617F* and *MPL* were negative in both the colonic MS and B-cell ALL (Fig. 2).

Amplicon-based NGS of a panel of 48 genes commonly mutated in hematologic malignancies was performed on the Ion Torrent PGM platform from unfractionated bone marrow DNA.

An inferred phylogenetic tree was constructed from NGS and karyotype data from bone marrow samples obtained during the evolution from ET to MDS, as well as from colon and pleural effusion samples obtained during blast transformation. The corresponding time-scale data points were aligned to reflect the hypothetical evolution of hematopoietic stem cells (HSC) most parsimonious with the sequencing and FISH findings reflected in Table 1. Note that between any two splits of the tree, the order of mutations and chromosomal deletions cannot be determined, and mutations are depicted sequentially for visual convenience (Fig. 2).

## Discussion and Conclusions

In this single case report, genomic reconstruction importantly revealed the presence of a large del(5q) in all three malignancies: ET, MS and B-cell ALL, suggesting a common cell of origin. In chronic phase MPN, this finding should inform physicians about the imminent likelihood for disease progression. Episomal reprogramming has identified del(5q) as an early cytogenetic lesion with the capacity to perturb genome stability and differentiation [6]. Larger del(5q) size has been correlated with higher mutation frequency [5], which in this case included the somatic driver mutations: *TET2, TP53*, *SF3B1*, and *ASXL1* at high allele frequency (Table 1). We propose that this combination led to HSC injury and disease progression through sub-clone branching (Fig. 2). The shared presence of *JAK2-V617F and TET2* mutations in ET suggests that “megakaryocytic branching” originated directly from the HSC as opposed to a more lineage-restricted progenitor [7]. The *JAK2-V617F* mutation confers a weak proliferative advantage to HSC and its absence in blast phases suggests MS and B-cell ALL did not emerge from this subclone.

Before blast conversion, the patient received lenalidomide, an immune modulator that yields cytogenetic remission by inhibiting growth of del(5q) progenitors without affecting other cells [4]. Clinically, lenalidomide improves survival and reduces transfusion requirements in patients with del(5q) MDS [4, 8]. Some reports suggest an association between lenalidomide therapy and transformation to more aggressive phenotypes, including a review documenting transformation of MPN to ALL [3]. Emerging data suggest that progression to AML in patients treated with lenalidomide is associated with karyotype complexity and clonal selection rather than a drug-mediated transformation [4,9]. Accordingly, in our case, bone marrow biopsies after lenalidomide therapy reveal absence of del(5q) clones, likely as a result of known suppression from drug. They also revealed a new 20q deletion (Table 1), which may portend the malignant potential of uninhibited clones, as evidenced later by its high expression in B-cell ALL.

Based on the ancestral reconstruction of genomic data modeled in Fig. 2, we hypothesize a mechanism for disease acceleration, whereby subclonal events with potential for blast conversion coexisted with but did not originate from *JAK2* p.V617F-positive ET. The presence of del(5q) and molecular abnormalities (*TP53*, *KMT2A*) in both MS and B-cell ALL (Fig. 2) suggest that “disease progression” originated from diversification of a pluripotent HSC capable of both myeloid and lymphoid differentiation [10, 11], thereby leading to two distinct leukemia initiating cells (LIC): one containing 17p deletion and the other 20q deletion. Interestingly, del(5q) was suppressed in bone marrow but not from extramedullary sites where the blast phase manifested, suggesting variable sensitivity of different clones to lenalidomide. The concurrent presence of *TP53* mutation in extramedullary sites [12] is known to confer a negative impact on survival and drug response to patients with del(5q) MDS treated with lenalidomide [7, 13]. Recent studies suggest that patients with high-risk MDS, characterized by unfavorable-risk cytogenetic abnormalities and/or *TP53* mutations, exhibit favorable clinical responses with robust mutation clearance when treated with hypomethylating agents (HMA) [14]. Unfortunately, HMA do not provide durable responses. A combination of azacytidine plus anti-CD47 monoclonal antibody is currently being investigated on *TP53*-AML with preliminary results showing an objective response in 71% of subjects and 48% complete remission [15].

We are limited in our ability to confirm the proposed order of pathogenic mutations. We lack banked bone marrow cells at all time points of disease evolution to demonstrate the proposed patterns of clonal progression. As this is a single case report, computer simulation of similar cases of accelerated phase ET would reaffirm our proposed model. However, collecting replicate cases is difficult given the rarity of the events described. Despite the limitations in our analysis, the ancestral tree in this case report highlights how the relative accessibility of NGS continues to improve our understanding of leukemogenesis, specifically, the predictive significance of large del(5q). It also has the capacity to inform therapeutic choices. Notwithstanding the presence of del (5q), recent studies support that HMA is a superior choice to lenalidomide under the clinical scenario described here. HMA combination regimens [15] currently being investigated may provide durable responses to patients with *TP53* mutations.

In conclusion, the advances in NGS technology have made it possible to generate a deep snapshot of the genetic composition of rapidly evolving tumor cells. The development and application of computational algorithms to harness NGS data for ancestral reconstruction could have a dramatic impact in how we practice medicine. Large scale simulation of similar cases to that described in this report would enhance our ability to predict disease progression. It would inform how a specific collection of molecular mutations would favor one therapeutic approach versus another. We propose exploring the use of computational algorithms to resolve patterns of clonal progression to enhance our understanding of leukemogenesis and the contribution of molecular targets, thereby leading to the development of more specific, tumor-tailored therapeutic interventions.

## Data Availability

The datasets generated and analyzed during the current study are available in the National Center for Biotechnology Information (NCBI) Sequence Read Archive (SRA) repository, accession number PRJNA727835 (http://www.ncbi.nlm.nih.gov/bioproject/727835). Mutational analyses by targeted sequencing panels are provided in the data table.

## Abbreviations

ALL: Acute lymphoblastic leukemia
AML: Acute myelogenous leukemia
CT: Computed tomography
ET: Essential thrombocythemia
FISH: Fluorescent in situ hybridization
HMA: Hypomethylating agents
HSC: Hematopoietic stem cell
LIC: Leukemia initiating cell
MDS: Myelodysplasia
MPO: Myeloperoxidase
MS: Myeloid sarcoma
NGS: Next generation sequencing
Ph-MPN: Philadelphia-negative myeloproliferative neoplasm
PMF: Primary myelofibrosis
PV: Polycythemia vera

## References

[1] Rampal R, Ahn J, Abdel-Wahab O (2014). Genomic and functional analysis of leukemic transformation of myeloproliferative neoplasms. Proc Natl Acad Sci U S A.

[2] Iurlo A, Cattaneo D, Gianelli U. Blast transformation in myeloproliferative neoplasms: risk factors, biological findings, and targeted therapeutic options. Int J Mol Sci 2019;20:10.3390/ijms20081839. 10.3390/ijms20081839 PMC6514804 31013941

[3] Alhuraiji A, Naqvi K, Huh YO, Ho C, Verstovsek S, Bose P (2017). Acute lymphoblastic leukemia secondary to myeloproliferative neoplasms or after lenalidomide exposure. Clin Case Rep.

[4] Giagounidis A, Mufti GJ, Fenaux P, Germing U, List A, MacBeth KJ (2014). Lenalidomide as a disease-modifying agent in patients with del(5q) myelodysplastic syndromes: linking mechanism of action to clinical outcomes. Ann Hematol.

[5] Stengel A, Kern W, Haferlach T, Meggendorfer M, Haferlach C (2016). The 5q deletion size in myeloid malignancies is correlated to additional chromosomal aberrations and to TP53 mutations. Genes Chromosomes Cancer.

[6] Hsu J, Reilly A, Hayes BJ (2019). Reprogramming identifies functionally distinct stages of clonal evolution in myelodysplastic syndromes. Blood.

[7] Noetzli LJ, French SL, Machlus KR (2019). New Insights Into the Differentiation of Megakaryocytes From Hematopoietic Progenitors. Arterioscler Thromb Vasc Biol.

[8] Lee JH, List A, Sallman DA (2019). Molecular pathogenesis of myelodysplastic syndromes with deletion 5q. Eur J Haematol.

[9] Pellagatti A, Jadersten M, Forsblom AM (2007). Lenalidomide inhibits the malignant clone and up-regulates the SPARC gene mapping to the commonly deleted region in 5q- syndrome patients. Proc Natl Acad Sci U S A.

[10] Xie W, Chen Z, Wang SA (2019). Lymphoblastic leukemia following myelodysplastic syndromes or myelodysplastic/myeloproliferative neoplasms. Leuk Lymphoma.

[11] Janssen JW, Buschle M, Layton M (1989). Clonal analysis of myelodysplastic syndromes: evidence of multipotent stem cell origin. Blood.

[12] Tang G, DiNardo C, Zhang L (2015). MLL gene amplification in acute myeloid leukemia and myelodysplastic syndromes is associated with characteristic clinicopathological findings and TP53 gene mutation. Hum Pathol.

[13] Mossner M, Jann JC, Nowak D (2016). Prevalence, clonal dynamics and clinical impact of TP53 mutations in patients with myelodysplastic syndrome with isolated deletion (5q) treated with lenalidomide: results from a prospective multicenter study of the german MDS study group (GMDS). Leukemia.

[14] Welch JS, Petti AA, Miller CA (2016). TP53 and Decitabine in Acute Myeloid Leukemia and Myelodysplastic Syndromes. N Engl J Med.

[15] Sallman D, Asch A, Kambhampati S, et al. The first-in-class anti-CD47 antibody magrolimab combined with azacitadine is well-tolerated and effective in AML patients: Phase 1b results. In: 62nd American Society of Hematology Meeting 2020;Session 613:Abstract 330 (oral).

